# Optimization of motion management parameters in a synchrotron‐based spot scanning system

**DOI:** 10.1002/acm2.12702

**Published:** 2019-09-20

**Authors:** Jedediah E. Johnson, Michael G. Herman, Jon J. Kruse

**Affiliations:** ^1^ Department of Radiation Oncology Mayo Clinic Rochester 200 First Street SW Rochester MN 55905 USA

**Keywords:** gating, interplay effect, particle therapy, repainting, respiratory motion management

## Abstract

**Purpose:**

To quantify the effects of combining layer‐based repainting and respiratory gating as a strategy to mitigate the dosimetric degradation caused by the interplay effect between a moving target and dynamic spot‐scanning proton delivery.

**Methods:**

An analytic routine modeled three‐dimensional dose distributions of pencil‐beam proton plans delivered to a moving target. Spot positions and weights were established for a single field to deliver 100 cGy to a static, 15‐cm deep, 3‐cm radius spherical clinical target volume with a 1‐cm isotropic internal target volume expansion. The interplay effect was studied by modeling proton delivery from a clinical synchrotron‐based spot scanning system and respiratory target motion, patterned from surrogate patient breathing traces. Motion both parallel and orthogonal to the beam scanning direction was investigated. Repainting was modeled using a layer‐based technique. For each of 13 patient breathing traces, the dose from 20 distinct delivery schemes (combinations of four gate window amplitudes and five repainting techniques) was computed. Delivery strategies were inter‐compared based on target coverage, dose homogeneity, high dose spillage, and delivery time.

**Results:**

Notable degradation and variability in plan quality were observed for ungated delivery. Decreasing the gate window reduced this variability and improved plan quality at the expense of longer delivery times. Dose deviations were substantially greater for motion orthogonal to the scan direction when compared with parallel motion. Repainting coupled with gating was effective at partially restoring dosimetric coverage at only a fraction of the delivery time increase associated with very small gate windows alone. Trends for orthogonal motion were similar, but more complicated, due to the increased severity of the interplay.

**Conclusions:**

Layer‐based repainting helps suppress the interplay effect from intra‐gate motion, with only a modest penalty in delivery time. The magnitude of the improvement in target coverage is strongly influenced by individual patient breathing patterns and the tumor motion trajectory.

## 1 INTRODUCTION

The potential of charged particle beams in providing highly conformal, targeted therapy has long been recognized.^1^, ^2^, ^3^ Compared with photon beams, charged particles exhibit a well‐defined penetration range in a patient, thus reducing unnecessary radiation dose distal to the intended target. Energy deposition rapidly increases near the end of a charged particle track at the location of the so‐called Bragg Peak, potentially allowing for a larger ratio of target to normal tissue dose.^4^

Technological advances have spurred the proliferation of proton therapy as an increasingly conventional treatment modality.^5^ Modern facilities almost exclusively feature pencil‐beam scanning,^6^ in which a small beamlet of fixed energy (corresponding to a specific depth) is scanned over the lateral extent of the target. Once the layer is completed, the system switches energy to a more proximal depth, and the target is again laterally scanned. This process is completed until the prescribed dose has been fully delivered. Treatment planning systems (TPS) optimize the number of energy layers, the positions of the beamlets in each layer, and the relative weightings of each spot delivery. Pencil‐beam scanning has advantages over the more traditional passively scattered approach including the ability to simultaneously shape both the proximal and distal boundaries of a target, the reduction of patient‐specific overhead in the form of custom apertures, and the ability to delivery intensity‐modulated proton therapy (IMPT).^4^ Along with these benefits, however, pencil‐beam scanning introduces the potential for deleterious interplay effects between the highly modulated delivery and a mobile target,^7^ whereas the time independence of passively scattered deliveries render these treatments much more robust to target motion.

The interplay effect in spot scanning has been shown to induce clinically relevant dosimetric defects in single fraction deliveries. This has been demonstrated both in general simulations^8^, ^9^ and in 4DCT studies on lung patients.^10^, ^11^ In this latter approach, the spots from a given field are divided temporally across all the 4DCT phases, dose is calculated on these phases, and the total dose is accumulated back to a reference phase. As spot sizes continue to decrease to achieve higher target conformality, the interplay effect is expected to increase in severity.^12^, ^13^

A number of techniques have been proposed to address the interplay effect. Beam gating, in which the beam is automatically enabled or terminated based on the continuous monitoring of a target motion surrogate, is a standard method of motion management in radiotherapy.^14^ It requires the placement of either internal or external fiducial markers,^15^, ^16^ corresponding real‐time tracking infrastructure, and upfront acquisition of 4D images at simulation. While gating has a history of successfully reducing the size of treatment volumes required to cover moving targets,^17^ suboptimal results are possible when it is applied to spot‐scanned proton treatments. Residual motion inside the gate can still disrupt dosimetric homogeneity,^18^ and complex target shapes and deliveries can render erratic results.^19^ Moreover, long treatment times are possible from the combined dynamics of beam‐off and accelerator recharge (for synchrotron systems) periods.^20^ Repainting, in which the treatment plan is subdivided and delivered in multiple iterations, provides an alternative to gating. Multiple delivery passes over the moving target are intended to smooth the dose by reducing the fractional effects of misplaced spots.^21^, ^22^, ^23^, ^24^ Unlike gating, repainting does not attempt to limit the magnitude of spot position deviations from their ideal/planned location in the target. Target tracking has also been investigated as a means of nullifying the interplay effect.^25^ While these techniques show promise, there remain substantial technical challenges and the potential to deliver significant unintended dose to surrounding normal tissue (or substantially lower dose to the target). Limitations of predictive tracking algorithms, anatomical heterogeneities upstream of the target, and relative motion between surrounding normal tissues and the target can all contribute to these effects.^26^, ^27^

With such a range of motion management options and associated configurable parameters, it is difficult to determine the optimal clinical solution for interplay effect reduction. Combined gating and repainting has been shown to offer added interplay reduction benefits in cyclotron delivery systems.^19^ As the severity of the interplay effect is dependent on delivery dynamics,^28^ however, it is important to characterize these effects and their associated countermeasures for different delivery systems. In this work, the relative effectiveness of gating, repainting, and combined gating/repainting is quantified for a representative spherical target and beam delivery using a synchrotron‐based system (Hitachi Ltd. Hitachi, Japan). Full three‐dimensional dose distributions on a quasi‐continuous mobile target in a water phantom were calculated and analyzed using dose volume histograms (DVH) metrics. Furthermore, the average and variability in expected dosimetric outcomes were determined by incorporating realistic target motion, modeled with a set of surrogate patient breathing traces, in multiple directions. Recommendations are suggested for the utilization of these motion mitigation strategies in pencil‐beam proton therapy.

## 2 METHODS

### 2.1 4**D Dose calculations**

Accurate calculation of dose to a moving target is a challenging problem, especially when coupled with the highly modulated delivery characteristics of spot‐scanned proton beams. The deployment of clinical tools has been limited by complexities associated with irregular, patient‐specific target motion, temporal beam delivery dynamics, and the high degree of manual intervention required when performing deformable image registration and dose accumulation. 4D dose calculation techniques potentially suffer from insufficient time resolution, which is limited by the number of reconstructed phases in a breathing cycle. Additionally, typical 4DCT scans only provide the motion trajectory of the target through a single, representative breathing cycle, thus neglecting cycle‐to‐cycle variability in both breathing amplitude and period.

To comprehensively study interplay effects and mitigation strategies amidst these complicating factors, an in‐house numeric simulation was developed to perform pencil‐beam proton dose calculations on moving targets. These three‐dimensional dose calculations were performed with high spatial resolution (0.75 mm) in a flat, homogeneous water phantom. This idealized geometry allows for the isolation of true interplay effects without convolution of independent effects such as range modulation due to mobile heterogeneities. The analytic calculation engine was based on models detailed in our TPS documentation^29^ (Eclipse, Varian), in which primary, secondary, and recoil particles are considered.^30^, ^31^ The depth dose was modeled using a corrected Bethe formula approach, and was parameterized by the incident proton energy and its intrinsic energy spread. Lateral scattering was governed by a double Gaussian dependence, which was fit to Moliere theory in water. Monte Carlo simulations of proton beams in water were used to fit the individual model coefficients for the highest spatial dose calculation accuracy. Beam divergence was modeled assuming a 245‐cm source‐to‐isocenter distance. The incident proton beams were modeled using symmetric, two‐dimensional Gaussian profiles in air. These energy‐dependent profiles were generated by Monte Carlo simulations of our spot scanning nozzle, and ranged between 3 and 6 mm σ in air. Total dose distributions are created by aggregating the modeled doses from each individual spot.

A test plan was created by delineating a 3‐cm radius clinical target volume (CTV) at 15‐cm mid‐depth. Isocenter was placed at the center of this target. A 1‐cm isotropic expansion was then applied, forming a 4‐cm radius internal target volume (ITV) to which a uniform 100 cGy was prescribed. This dose was intended to represent one field of a typical two field, 200‐cGy treatment fraction. No additional margin was added to account for setup uncertainties. As our motion simulation package does not include an optimization engine, the commercial TPS was used to define the spot positions and weights necessary for uniform coverage of the ITV. A spot spacing of 2.98 mm was chosen to match the default TPS spot spacing for this target configuration. After the spot positions and weights were determined, they were entered into our 4D dose simulation.

**Figure 1:**
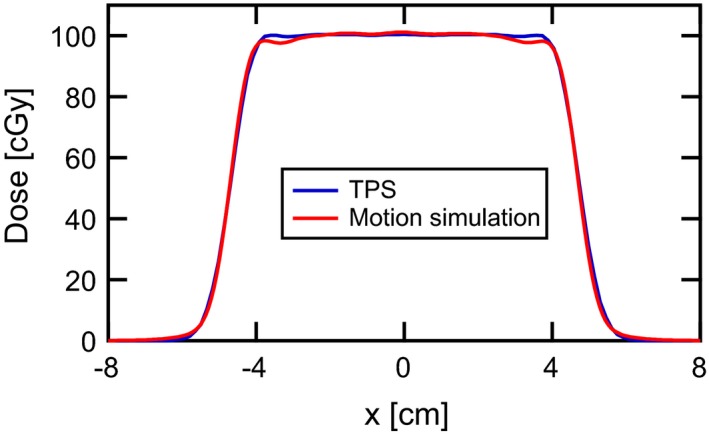
Calculated dose profile comparison. Beam’s eye view profiles through the center of the CTV demonstrate excellent agreement between the TPS and our motion simulation in the case of a static target. CTV, clinical target volume; TPS, Treatment planning system.

Before proceeding with mobile targets, the dose calculation engine associated with our motion simulation was validated against the TPS. A comparison of dose profiles, which are perpendicular to the beam’s eye view and pass through the center of the ITV, is shown in Figure 1. Excellent quantitative agreement was demonstrated throughout the entire spherical ITV, where the dose accumulation algorithm operates. After this initial validation, all subsequent dose/motion calculations were performed and analyzed entirely in our 4D motion simulation.

### 2.2 Target motion models

Our 4D dose calculation has the capability of simulating arbitrary target motion in three dimensions. In this study, one‐dimensional motion trajectories were analyzed independently. The trajectories considered were confined to the plane orthogonal to the central axis of the individual pencil beams. Directions in this plane can be referenced to the primary pencil‐beam scanning axis. In our system, the beam rasters back and forth in this direction as it paints a given energy layer, only making a perpendicular step to the next row of spots between subsequent sweeps. The two separate target motion directions considered in this work were “parallel” and “orthogonal” to this beam scanning axis in the plane transverse to the beam’s eye view.

**Figure 2:**
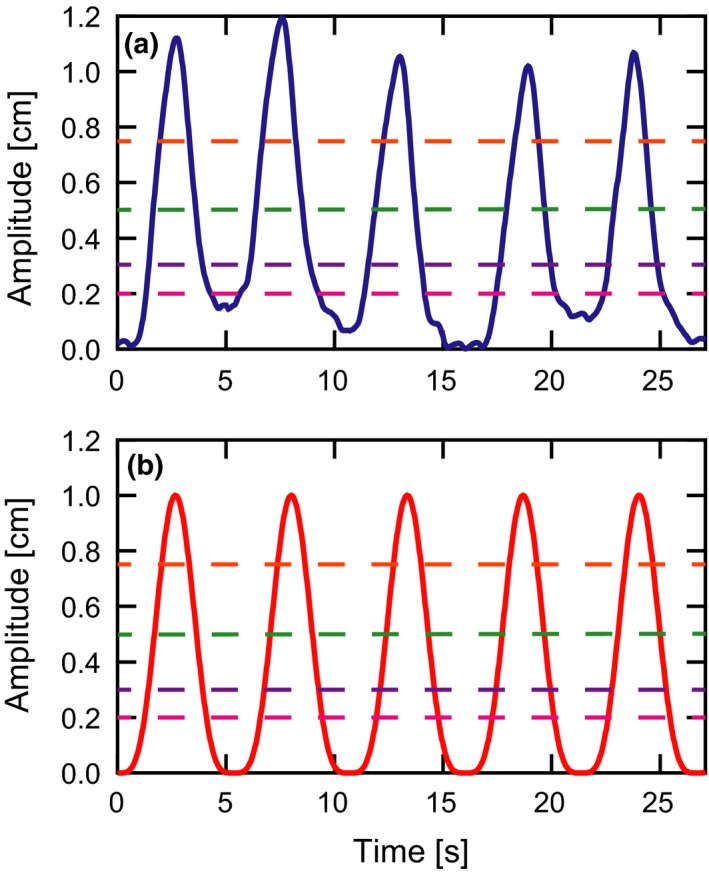
Target motion models. (a) Motion models acquired by tracking the position of target surrogates during clinical 4DCT procedures contain irregularities in both amplitude and period. (b) For comparison, an idealized $sin^{4}\left(\omega t\right)$ function is sometimes used as a model for respiratory motion. The dashed lines at 0.75 cm (orange), 0.5 cm (green), 0.3 cm (purple), and 0.2 cm (magenta) represent different amplitude gate thresholds used in delivery simulations with target motion.

Irregular breathing models were chosen to simulate target motion. In addition to the more realistic nature of this irregular motion, idealized periodic motion can lead to resonant interference effects in the delivered dose distribution.^21^ These effects are damped by cycle‐to‐cycle breathing variability. Thirteen distinct patient breathing traces were used to represent a spectrum of target motion patterns. These motion traces were acquired during clinical 4DCT scans using the Varian Real‐time Position Management (RPM) system. The position of an infrared‐reflecting surrogate marker, which is placed near the abdomen, was tracked by the system and saved for later analysis. Although this external surrogate is not rigidly linked with internal targets, the approximation was made to perfectly correlate the markers with target motion. The traces were first smoothed to reduce measurement‐related noise using a second order Savitzky–Golay filter with a moving span of 1 s. To standardize the nominal motion amplitude among the 13 patients, an overall scale factor was applied such that the resulting root mean square (RMS) amplitude of each trace was $\frac{1}{2\sqrt{2}}$ cm, consistent with a sinusoidal wave of 1‐cm peak‐to‐peak amplitude. Each trace was then systematically shifted so that its quiescent minimum nominally lined up with the other breathing traces at a common baseline coordinate, y = 0 cm. Figure 2(a) shows an example section of a patient breathing trace standardized in this manner. Irregularities can be visualized in the breathing amplitudes, baselines, and periods. An idealized, $sin^{4}\left(\omega t\right)$ model is shown for comparison in part (b).

### 2.3 Delivery timing parameterization

The temporal characteristics of the proton beam delivery strongly influence interplay effects, and vary from system to system. The parameters used in this work, shown in Table I, were modeled after those associated with our synchrotron‐based accelerator (Hitachi ProBeat‐V). The synchrotron spill rate, charge capacity and hold time, scan rates, and spot verification times are specified. Buffer periods, which account for latencies between gate and beam on/off signals, are also tabulated. The precise specification of these parameters, along with the target motion, allows for quasi‐continuous dose accumulation simulations. The delivery is simulated spot by spot, each spot being placed at the location corresponding to its temporal midpoint. This approximation is valid because the longest spot delivery times (~ 7 msec) are orders of magnitude shorter than typical breathing periods (~ 5 s).

### 2.4 Motion management techniques

**Figure 3:**
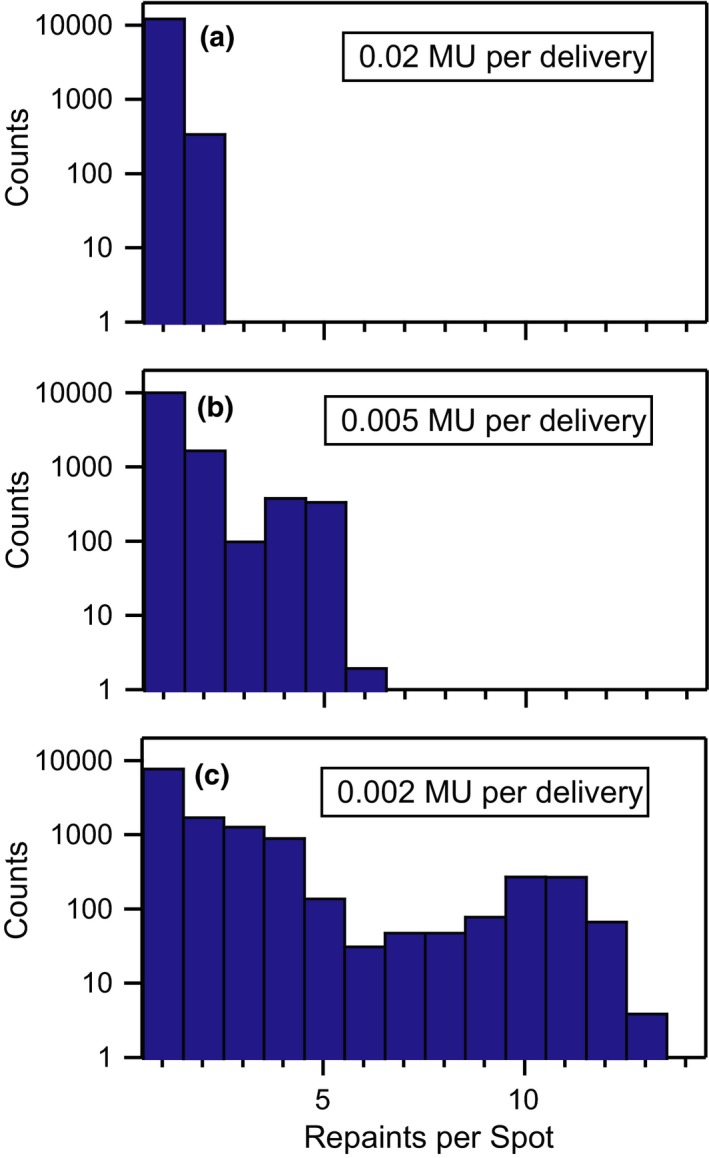
Spot delivery histograms. The distribution of deliveries per planned spot during maximum‐MU repainting is shown on a logarithmic scale for (a) 0.02, (b) 0.005, and (c) 0.002 MU per delivery. As expected, decreasing the maximum MU per spot shifts the distributions towards a higher number of deliveries.

Each of the 13 motion patterns was initially simulated without any motion mitigation strategy. Four successively smaller amplitude gate levels (0.75 cm, 0.5 cm, 0.3 cm, 0.2 cm shown in Figure 2) were then utilized to improve the dosimetric plan quality. As an alternative to gating, maximum‐MU layer‐based repainting was also investigated. In this technique, the MU in each individual spot delivery is capped at a specified maximum MU. A given energy layer is repeated multiple times — each spot receiving at most the maximum MU — until the total prescribed MU of each spot is delivered. The only exception to the maximum MU limit occurs when the any remaining MU are less than the synchrotron’s minimum deliverable MU (0.001 MU in this work). In this event, the MU of the preceding spot is allowed to exceed (by the remainder) the maximum to prevent an undeliverable scenario. The synchrotron only switches to the next energy layer after all the prescribed MU in a given layer have been delivered. This maximum‐MU form of layer‐based repainting was chosen because it is supported by our commercial TPS and does not require rapid energy changes. Maximum MU thresholds of 0.02, 0.015, 0.01, 0.007, 0.005, 0.004, 0.003, and 0.002 MU were all simulated. Figure 3 shows histograms of the number of deliveries for each of the 12936 spots, assuming maximum MU values of 0.02 (a), 0.005 (b), and 0.002 MU (c). Lastly, the combination of gating and maximum‐MU repainting was considered. All combinations of maximum MU thresholds and gate levels (except the smallest 0.2‐cm gate) were simulated.

### 2.5 Dosimetric analysis

In each case, dose volume histograms (DVH) were computed for the target volume. The information contained in the DVH curves was distilled into a more compact form by computing both the target coverage (V97%) and homogeneity index (D5% ‐ D95%) of each curve. Both the average and standard error of the mean across the 13 breathing traces were then calculated for each motion mitigation technique. To provide a context for useful comparison between techniques, the total treatment delivery time of each simulation was also calculated and averaged across the 13 breathing traces. This provides a standard metric to quantify the temporal cost associated with decreasing gate windows and increased levels of repainting, allowing for an analysis of the tradeoffs between plan quality and treatment delivery time. This in turn facilitates the ultimate goal of identifying the technique which provides the highest motion mitigation effectiveness and efficiency.

## 3 RESULTS

**Figure 4:**
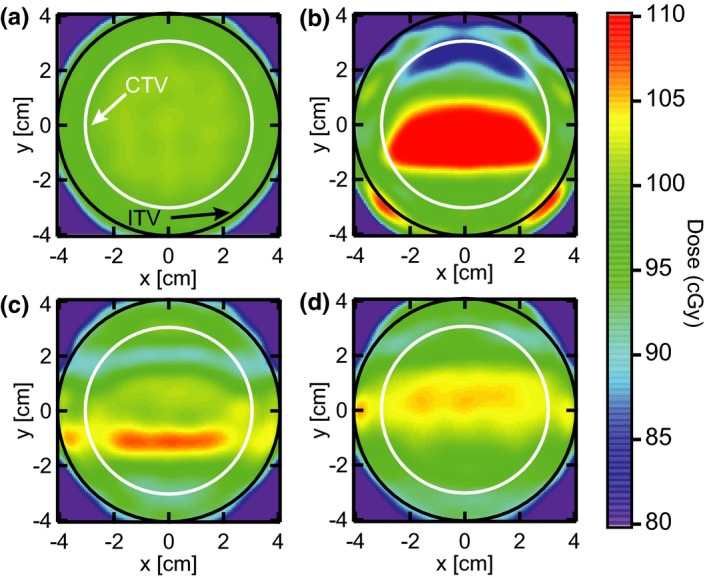
Beam’s eye view dose planes. An example of dose planes through the center of the CTV are shown for (a) the static target, (b) target motion without motion compensation, (c) target motion with a gate threshold of 0.3 cm, and (d) target motion with the same gate and maximum‐MU repainting with 0.005 MU per spot. The target motion is based off a representative patient breathing trace which has been scaled to 1‐cm nominal amplitude. The target moves in the plane of the beam’s eye view, orthogonal to the primary scanning axis of the proton delivery system. CTV, clinical target volume.

Examples of beam’s eye view dose planes through the center of the CTV are presented in Fig. 4. Part (a) shows the uniform dose distribution in the absence of target motion, while (b) is the result of orthogonal target motion for one particular patient breathing trace. The motion creates large regions of substantial over‐ and under‐dose in the CTV. With the introduction of a 0.3‐cm gate level (c), these effects are greatly diminished. Combining this gate window with 0.005 MU/spot maximum‐MU repainting (d) further reduces the magnitude and size of the over‐ and under‐dosed regions.

**Figure 5:**
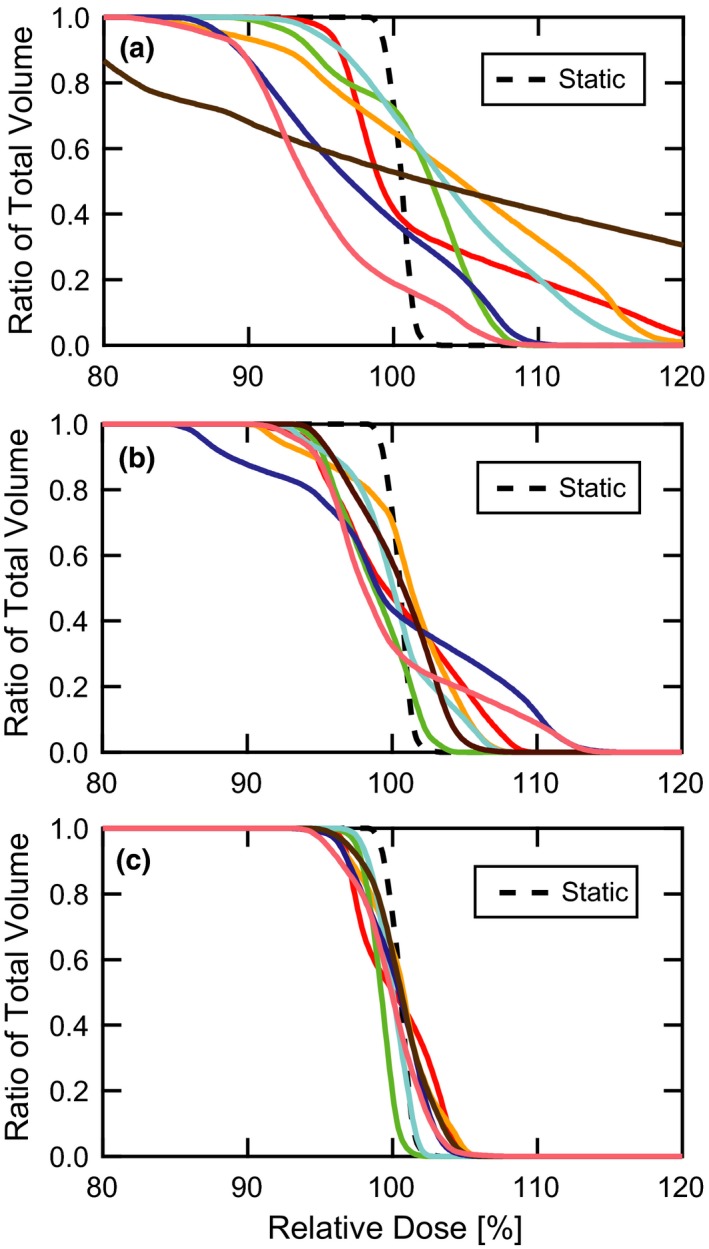
DVH comparisons. DVH curves are shown for (a) target motion without motion compensation, (b) target motion with a gate threshold of 0.3 cm, and (c) target motion with a gate threshold of 0.3 cm and maximum‐MU repainting with 0.004 MU per spot. The plots contain seven distinct patient breathing patterns along with the static target for reference. The target moves in the plane of the beam’s eye view, orthogonal to the primary scanning axis of the proton delivery system. DVH, dose volume histograms.

Figure 5 shows DVH curves corresponding to seven patient breathing traces with orthogonal target motion. The static DVH is also included for comparison. When no motion management is employed (a), the DVH curves generally exhibit unacceptably low target coverage along with high dose tails. In addition, there is a large degree of variability between the traces, making it difficult to predict how much degradation can be expected from target motion. Even though both the nominal amplitude (~ 1 cm) and motion direction are common among these traces, patient‐specific differences in breathing period and other irregularities lead to a wide range of erratic outcomes. The resulting DVH curves when gating (0.3‐cm gate) and combined gating and repainting (0.3‐cm gate with 0.004 MU/spot maximum‐MUY repainting) are applied to the same motion patterns are shown in parts (b) and (c). As expected, an improvement is apparent in both the average plan quality and trace‐to‐trace consistency, particularly in the case of combined gating and repainting (c). Plans which more closely resemble the DVH of the static CTV, thus making them candidates for clinical acceptability, were achieved.

**Figure 6:**
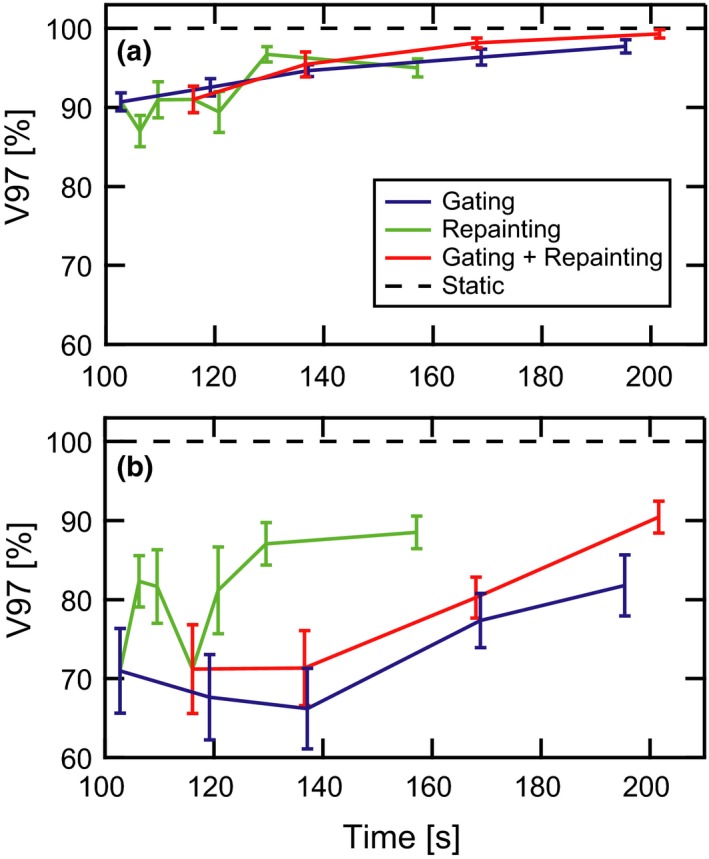
Target coverage vs delivery time. The average CTV coverage (V97%) and standard error of the mean across 13 distinct motion patterns are plotted against average delivery time. The target moves both (a) parallel to and (b) orthogonal to the primary scanning axis of the proton delivery system in the plane of the beam’s eye view. Curves are shown for the static target (black dashes), gated delivery (blue line), maximum‐MU repainting (green line), and combined gating and maximum‐MU repainting (red). CTV, clinical target volume.

The average CTV target coverage and standard error of the mean are plotted vs the average delivery time in Fig. 6 for both parallel (a) and orthogonal (b) target motion. Each plot consists of four series, including the static target coverage, gating only (variable gate level), repainting only (subset of variable maximum MU values), and gating combined with repainting (variable gate level with 0.005 maximum MU). The data point at the shortest delivery time (t = 102 s), common to both the gating only and repainting only series, corresponds to the absence of any motion mitigation. Comparison of the parallel and orthogonal target motion plots reveals significantly less target coverage for target motion orthogonal to the beam scanning axis. The gating and gating + repainting curves generally trend toward higher target coverage with increased delivery time. The associated error bars, which can be viewed as representing the variability in potential outcomes as a function of patient breathing differences, also generally decrease with increasing time. With the exception of the small time points in part (a), there is a general shift towards improved target coverage in the combined gating and repainting curve with respect to the gating‐only curve. This is also accompanied by smaller error bars, especially at the longer delivery times. The repainting‐only curves show volatile behavior in the large maximum MU/low delivery time region, stabilizing with decreasing error bars at larger time values. For orthogonal motion, repainting only appears to provide the most efficient benefit for modest delivery time increases.

**Figure 7:**
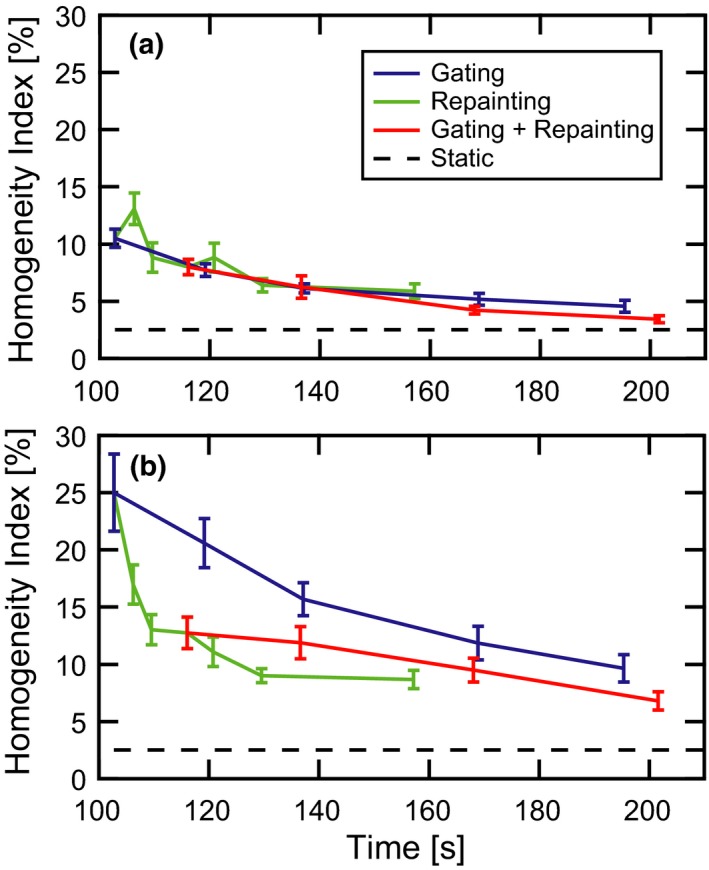
Homogeneity index vs delivery time. The average CTV homogeneity index (D95% – D5%) and standard error of the mean across 13 distinct motion patterns are plotted against average delivery time. The target moves both (a) parallel to and (b) orthogonal to the primary scanning axis of the proton delivery system in the plane of the beam’s eye view. Curves are shown for the static target (black dashes), gated delivery (blue line), maximum‐MU repainting (green line), and combined gating and maximum‐MU repainting (red). CTV, clinical target volume.

In a format analogous to Fig. 6, the homogeneity index of the CTV for parallel (a) and orthogonal (b) motion is presented in Fig. 7. There is a general trend towards better dose homogeneity and lower patient‐to‐patient variability with longer delivery times. Orthogonal target motion is again associated with greater plan degradation than parallel motion. For parallel motion, the three curves are nearly coincident for treatment times below 150 s, albeit with erratic behavior in the repainting‐only curve. At longer delivery times, a shift toward greater dose homogeneity and smaller error bars is visible in the combined gating and repainting vs gating‐only curve. This shift and error bar reduction is also visible across the full range of delivery times for orthogonal target motion. Repainting alone also provides the most dramatic reduction of dose heterogeneity for small deliver time increases.

**Figure 8:**
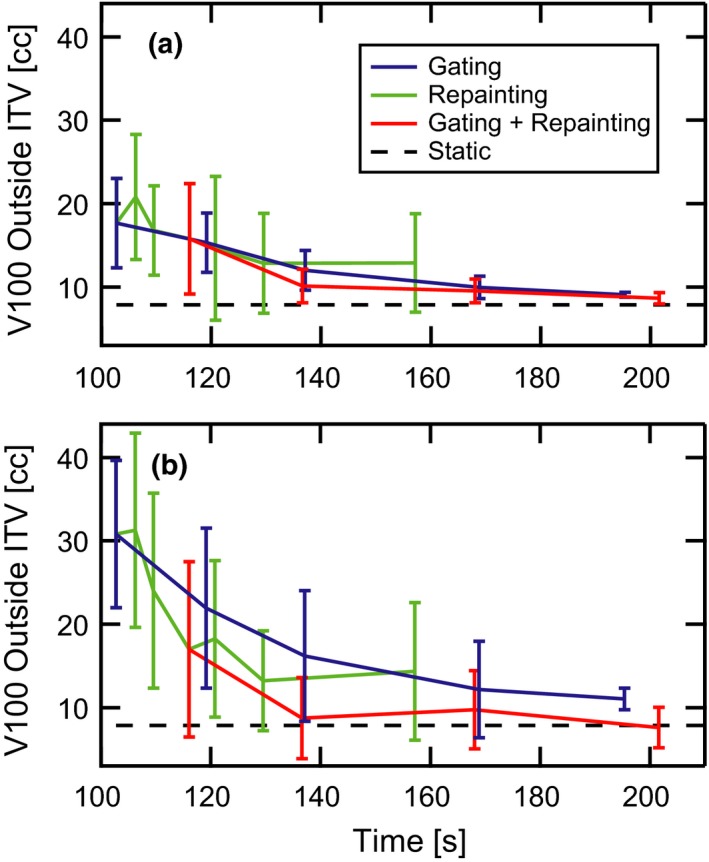
High dose spillage vs delivery time. The average volume receiving prescription dose outside the ITV and standard error of the mean across 13 distinct motion patterns are plotted against average delivery time. The target moves both (a) parallel to and (b) orthogonal to the primary scanning axis of the proton delivery system in the plane of the beam’s eye view. Curves are shown for the static target (black dashes), gated delivery (blue line), maximum‐MU repainting (green line), and combined gating and maximum‐MU repainting (red). ITV, internal target volume.

The volume outside the ITV receiving prescription dose is plotted in Fig. 8 as a function of delivery time for parallel (a) and orthogonal (b) target motion. This dose spillage decreases with increasing delivery times for each motion management strategy. Although differences in the data sets are partially obscured by the large error bars, the average dose spillage across all delivery times is lowest for combined gating and repainting. A significant feature of the plot is the reduction in the patient‐to‐patient dose spillage variability represented by the error bars for techniques which include gating and longer delivery times. Even for the longest delivery times in the repainting‐only traces, the error bar magnitudes remain relatively large.

## 4 DISCUSSION

Without the use of any motion mitigation technique, it is qualitatively evident from Fig. 5 that the interplay effect produces unacceptable plan quality degradation for nominal 1‐cm orthogonal target motion. This worst‐case conclusion must be tempered somewhat when considering different motion directions. For parallel motion, the average target coverage V97% was greater than 90%, with a heterogeneity index slightly above 10%. In certain circumstances, these dose metrics may be considered acceptable, depending on a number of factors including the ultimate clinical objective of the treatment plan. Although these average metrics may seem acceptable, however, the stochastic variability in plan outcome with individual, fraction‐specific breathing characteristics may be excessive. This is particularly true in the case of hypofractionated treatment regimens, where the total composite dose could be dominated by individual fractions in which the interplay effect is strongly manifest. Conventional fractionation schemes and the use of multiple treatment fields counter this effect by increasing the number of beam deliveries, which creates smoother composite dose distributions by means of averaging across a larger variety of breathing patterns. Nevertheless, there are still perils to reliance on these effects, as tumorcidal cell killing is a function of the individual dose per fraction. While the total composite dose provides a convenient summary of a treatment course, it does not adequately convey the radiobiological differences associated with fraction‐to‐fraction dose variations. Consequently, motion management techniques should aim to reduce inter‐fraction dose variability, represented by the size of the error bars, as much as possible.

Repainting alone for small delivery time increases provides the greatest benefit vs gating‐based techniques for orthogonal motion, whereas the trend is more unpredictable and erratic for parallel motion. These issues appear to be less severe for the two longest delivery time data points, which represent 0.003 and 0.002 MU per spot. While the effectiveness of repainting in smoothing dose heterogeneities was expected, its comparability to gating for restoration of target coverage may be less intuitive as repainting techniques do not inhibit the delivery of spots outside the target. This effect is responsible for the clear inferiority of repainting alone compared to gating with respect to high dose spillage outside the ITV. The large error bars associated with the repainting‐only curve indicate a non‐negligible probability of a large hot spot outside of the ITV.

Without restrictions on minimum deliverable MU, gating allows for larger degrees of target motion mitigation, as reflected by the much longer delivery times typical of small gate thresholds. This practical limitation is the tradeoff for the increased plan quality associated with tighter gate thresholds. For longer delivery times, gating alone produces superior average plan quality as measured by target coverage, target homogeneity, and dose spillage when compared with repainting alone. Similarly, the combination of gating and MU repainting with 0.005 MU per spot results in systematically better plan quality than gating alone for a fixed delivery time across the entire range of treatment delivery times. Phrased another way, the combination of gating and MU repainting reduces the total delivery time for a specifically targeted plan quality, allowing for more efficient utilization of the treatment room. This is illustrated by an example taken from the data in Figs. 6 and 7. For orthogonal motion, the average target coverage and homogeneity index are 71.0 ± 5.4% and 25.0 ± 3.4% (1 σ of the mean), respectively, in a delivery time of 102.8 s with no motion mitigation. When a small gate window is used, these values improve to 81.8 ± 3.9% and 9.7 ± 1.2 in an average delivery time of 195.4 s. When repainting with 0.005 MU per spot is incorporated with a relaxed gate threshold, the coverage and heterogeneity remain close to the gating only case at 80.2 ± 2.0% and 9.5 ± 1.0%, respectively, but in an average delivery time of only 168.1 s. Ultimately, the appropriate balance between plan quality and delivery time should be a decision based on the clinical objectives and patient throughput.

**Table 1:** Delivery timing parameters. Dose distributions resulting from interplay effects are sensitive to the timing characteristics of the delivery system. The parameters used in this work were modeled after our synchrotron system.

Timing parameter	Value
Energy switching/Charge refill time	2.3 s
Synchrotron charge capacity	2 nC
Synchrotron charge hold time	8 s
Synchrotron charge spill rate	0.5 nC/s
Beam scan rate	10 m/s
Beam orthogonal step rate	6 m/s
Spot position verification time	2.5 ms
Gate off buffer	40 ms
Gate on buffer	200 ms

A study performed by Schätti et al.^19^ indicated that combined gating and repainting increased the safety and robustness of spot scanning motion management when compared to gating alone. The simulation aspect of this work was performed by assuming a cyclotron‐based delivery, an irregular breathing model obtained from a single patient, and dose metric comparisons were based on analysis of a single two‐dimensional plane near the central lateral plane of the target. As the magnitude of the interplay effect is dependent on machine delivery characteristics such as spot size, beam current, energy switching times, and scanning speed, it is valuable to characterize these effects in different delivery systems and under a variety of clinical scenarios. In this work, we provide data to aid in the clinical implementation of a motion management program for a spot scanning, synchrotron‐based Hitachi delivery system. Additionally, by incorporating statistical analysis on the results obtained from 13 irregular patient breathing models, motion management parameters could be more optimally tuned to account for the natural spread of patient breathing characteristics. Finally, as interplay is dependent on target depth, a single two‐dimensional plane may not capture all relevant effects. We compliment previous work in two dimensions by extending our analysis to the full three‐dimensional target volume.

## 5 CONCLUSION

A quasi‐continuous simulation of arbitrary target motion in a water phantom has been developed for spot scanning proton plans delivered using a Hitachi synchrotron‐based system. This has enabled calculation of the 4D dose accumulation for a variety of circumstances, including different target motion directions, patient breathing patterns, and motion mitigation strategies. Plan quality, as measured by target coverage, target homogeneity, and prescription dose spillage outside the ITV, is substantial better for target motion parallel to the primary pencil‐beam scanning axis as opposed to the case when this scanning axis is rotated 90 degrees. Averaged over 13 patient breathing traces normalized to nominal 1‐cm peak‐to‐peak amplitude, the combination of gating and layer‐based repainting allows for a shorter treatment delivery time and/or superior average plan quality with less variability when compared with gating or repainting alone. Clinical constraints and individualized objectives should be considered when optimizing the specific gate and repainting parameters. Looking ahead, our simulation infrastructure will allow for comparison with measured dose distributions, as well as investigation into additional dependences such as target size, prescription dose, breathing amplitude, and complex three‐dimensional motion patterns.

## CONFLICT OF INTEREST

No conflicts of interest.
